# Quantifying Stress and Relaxation: A New Measure of Heart Rate Variability as a Reliable Biomarker

**DOI:** 10.3390/biomedicines13010081

**Published:** 2025-01-01

**Authors:** Emese Rudics, András Buzás, Antónia Pálfi, Zoltán Szabó, Ádám Nagy, Emőke Adrienn Hompoth, József Dombi, Vilmos Bilicki, István Szendi, András Dér

**Affiliations:** 1 Doctoral School of Interdisciplinary Medicine, University of Szeged, H-6720 Szeged, Hungary; rudicsemese8@gmail.com; 2 Institute of Biophysics, HUN-REN Biological Research Centre, H-6701 Szeged, Hungary; buzas.andras@brc.hu; 3 Department of Software Engineering, University of Szeged, H-6720 Szeged, Hungary; palfi@inf.u-szeged.hu (A.P.); szaboz@inf.u-szeged.hu (Z.S.); adam.nagy@inclouded.hu (Á.N.); hompothemoke@gmail.com (E.A.H.); bilickiv@inf.u-szeged.hu (V.B.); 4 HUN-REN-SZTE Research Group on Artificial Intelligence, Institute of Informatics, University of Szeged, H-6701 Szeged, Hungary; dombi@inf.u-szeged.hu; 5 Department of Psychiatry, Kiskunhalas Semmelweis University Teaching Hospital, H-6400 Kiskunhalas, Hungary

**Keywords:** heart rate variability, biofeedback training, stress, normalized variability, master curve, relaxation

## Abstract

**Background/Objectives:** For the rapid, objective characterization of the physiological stress response, there is currently no generally recognized standard. The stress measurement methods used in practice (e.g., for psychological measures of stress) are often subjective, or in the case of biological markers (e.g., cortisol, amylase), they usually require a blood test. For this reason, the use of heart rate variability (HRV) to characterize stress has recently come to the fore. HRV is the variability in the length of heartbeat intervals, which indicates the ability of the heart to respond to various physiological and environmental stimuli. However, the conventional HRV metrics are not corrected for heart rate dependence; hence, they fail to fully account for the complex physiology of stress and relaxation. In order to remedy this problem, here we introduce a novel HRV parameter, the normalized variability derived from an RMSSD “Master Curve”, and we compare it with the conventional metrics. **Methods:** In Study 1, the relaxation state was induced either by heart rate variability biofeedback training (N = 21) or by habitual relaxation (N = 21), while in Study 2 (N = 9), the Socially Evaluated Cold Pressor Test and the Socially Evaluated Stroop Test were used to induce stress in the subject. For a statistical evaluation of the data, the Kolmogorov–Smirnov test was used to compare the distributions of mean HR, log(RMSSD), log(SDNN), and normalized variability before, during, and after relaxation and stress. **Results:** The results of this study indicate that while log(RMSSD) and log(SDNN) did not change significantly, the normalized variability did undergo a significant change both in relaxation states and in stress states induced by the Socially Evaluated Cold Pressor Test. **Conclusions:** Overall, we suggest this novel type of normalized variability ought to be used as a sensitive stress indicator, and in general, for the characterization of the complex processes of the vegetative nervous system.

## 1. Introduction

Heart rate variability (HRV), the measure of beat-to-beat variation in interbeat intervals, is considered an indicator of the autonomic nervous system (ANS), and it represents the variation in beat-to-beat time intervals over a period of time [1]. HRV may be influenced by several nonmodifiable factors (age, gender, ethnicity, genetics, circadian rhythm) and modifiable factors: physiological and pathological factors (posture, respiration, endocrine factors, inflammation, infection, neurological disorders, cardiovascular diseases), lifestyle factors (alcohol, tobacco and drug consumption, physical activity, meditation), environmental factors (social stress, noise, temperature, etc.), and neuropsychological factors (mental stress, depression) [1,2,3,4]. The changes in heart rate (HR) also affect HRV, since a decrease in HR results in an increase in HRV, and conversely, an increase in HR results in a decrease in typical HRV parameter values, such as those for SDNN or RMSSD [5,6,7]. Since physical activity and posture influence both HR and HRV [2,4,5,8], according to the measurement protocol elaborated by the joint European and American task force [9], a fixed-position registration of the RR time series is preferred, where patients are kept stationary during HRV monitoring [9], which, however, complicates the examination in experimental situations that require movement. Hence, revealing the intricate relationship between HRV and HR has become increasingly imperative. There are a large number of publications discussing the problem of the HR dependence of crucial HRV parameters (see, amongst others, the publications of and the debates between the Billmann and the Boyett groups [5,6,7,10,11,12,13]), but, not knowing the exact function that describes the relationship between HRV and HR, there has been no precise way to define a single “heart rate-corrected” HRV parameter that could properly account for the HR-dependence (similar to, say, how the heart rate-corrected QT interval, “QTc”, is derived [14]).

To solve this problem, we recently suggested recording signals over a several-hour-long period under natural, daily routine circumstances by a wearable ECG device (i.e., Holter monitor sessions, where otherwise no special measuring conditions are required), and used a nonlinear evaluation method based on Bland–Altman-like plots, to derive a reference curve from these data, called the “Master Curve” (MC), that appeared to be rather conservative for each individual from the daily to monthly scales [15]. In that paper, we also suggested that the momentary deviations detectable on the minute scale from MC could potentially be usable as a measure of changes in the dynamics of the heart rhythm, accompanying temporary deviations from the balance of the autonomic nervous system. The present paper is the first application of this concept for stress- and relaxation-related physiological changes.

HRV analysis is frequently used in stress and relaxation studies [16,17,18], and the HRV parameters normally used here are frequency- and time-domain analyses, or nonlinear methods [1,19]. These parameters (such as RMSSD and SDNN in the time domain and the low- (LF) and high-frequency (HF) components in the frequency domain) are statistically defined from the variability in beat-to-beat intervals [20], and their value is significantly influenced by the effect of physical activity on the heart rate [5,8,21]. According to the literature [22], during an acute stress response, when the heart rate increases due to the increased activity of the sympathetic nervous system, both RMSSD and SDNN decrease [10,23]. In contrast to the stress response, during relaxation, the activity of the parasympathetic nervous system increases, resulting in a decrease in the heart rate [24]. Among others, heart rate variability biofeedback (HRV BF) training is an effective stress reduction method both after long-term practice [16,25] and after a single session of training [17,26].

During HRV BF training, participants breathe at a specific breathing frequency, called the resonance frequency. Resonance frequency individually varies, but in all cases, it is approximately around 0.1 Hz. By breathing at the resonance frequency, the heart rate and the respiration rate oscillate in a 0° phase relationship, and the respiratory sinus arrhythmia (heart rate increases when breathing in and decreases when breathing out) is maximized. In addition, the synchronization of the heart rate and the respiration rate by breathing on the resonance frequency stimulates the baroreflex and its receptors, the baroreceptors (stretch receptors in the carotid artery and the aorta). Baroreceptors detect changes in blood pressure by sensing the stretch of the arteries that varies according to changes in pressure in the arteries. When blood pressure increases and baroreceptors detect it, the baroreflex immediately decreases the heart rate and vice versa; when the blood pressure decreases, the system increases the heart rate. By breathing at the resonance frequency, the blood pressure and the heart rate oscillate in a 180° phase relationship. Slow-paced breathing at the resonance frequency, by maximizing respiratory sinus arrhythmia and stimulating baroreflex, increases HRV [24,27,28].

Lin and colleagues [17] found that during HRV BF training, SDNN and RMSSD were higher than before and after the training. Furthermore, after the HRV BF training SDNN remained higher compared to the baseline level of SDNN. In general agreement with the results of Lin et al. [17], others found an increase in SDNN 12 weeks after the beginning of HRV BF training [16]. In stark contrast, Park et al. [29] found no significant changes in RMSSD before and after several weeks of HRV BF training, and according to the study of Deschodt-Arsac et al. [30], there was no significant change between baseline RMSSD, RMSSD during stress exposure after several weeks of practicing HRV BF, and RMSSD in the resting period after several weeks of HRV BF training. However, in the control group not practicing HRV BF training, the RMSSD of the subjects significantly decreased during stress exposure compared to the baseline and resting periods.

To be able to describe the stress responses of HRV independently of the actual HR, here we applied the new method described in [15], and established an HRV(HR) Master Curve for each individual (for more details, see Section 2.3). MC represents a person-specific measure of HRV that is stable over a long time, but short-term fluctuations on the minute scale do occur. Hence, the momentary state of a person can be represented by these short-time deviations from the MC, meaning that the HRV activity of the momentary state is different from the average HRV of the person at the actual HR. Using MC as a normalization baseline, it is possible to filter out the disturbing effect of confounding variables (e.g., physical activity, posture) from HRV results [15], and in this way derive HR-corrected HRV values. The specific aim of this study was to compare stressed and relaxed states using the consensus-based HRV parameters RMSSD and SDNN, and to introduce a novel HRV parameter valid for the entire HR range, called the normalized variability, obtained by the normalization of the actual HRV parameter using the MC. We found it to be suitable for quantifying and discriminating stress and relaxed states independently of changes in the instantaneous HR. Here, we present two studies. In Study 1, stress was alleviated with heart rate variability biofeedback training and habitual relaxation, while in Study 2 stress was provoked using the Socially Evaluated Cold Pressor Test [31] and with a newly developed stress-induction method, called the Socially Evaluated Stroop Test.

We found that normalized variability decreases in relaxed states and increases during stress, whereas the consensus-based RMSSD and SDNN might indicate a contrary change following the change in HR, i.e., an increase during relaxed states and decrease during a stress state, or it might not change significantly. We expect that by using normalized variability in the evaluation of tests, we will better understand the physiology of stress and relaxation, as well as the interaction between the sympathetic and parasympathetic nervous systems.

## 2. Materials and Methods

### 2.1. Study 1: Relaxation Experiments

#### 2.1.1. Participants

Thirty-three healthcare professionals of the Semmelweis Hospital in Kiskunhalas, the Teaching Hospital of the University of Szeged, were recruited for this study. A within-subject design was utilized, so all the participants underwent both HRV BF and relaxation sessions. Participants’ HRV data sets were separated into two groups, namely the HRV BF group and relaxation group, based on the research conditions. Out of the thirty-three participants, both the HRV BF and relaxation HRV data of ten participants were excluded due to inappropriate data, and after further data cleaning, two more participants’ relaxation HRV data and another two participants’ HRV BF data were excluded. The final sample size consisted of an HRV BF group with N = 21 (18 women, 3 men) with a mean age of 44.29 years (SD = 9.12 years, range: 26.00–58.00) and a habitual RLX group N = 21 (17 women, 4 men) with a mean age of 44.71 years (SD = 9.63 years, range: 26.00–59.00).

We did not identify any participant with mental disorders during the MINI Interview, but as two of our participants had been diagnosed earlier with mental disorders (major depression and generalized anxiety disorder), both were receiving medical treatment and they were medically stable.

Informed consent was obtained from the participants before data collection. This study was approved by the local ethics committee at the National Public Health Center (6860-12/2022/EUIG). Financial compensation was provided for participants for their contributions to the study.

#### 2.1.2. Data Collection

The Mini International Neuropsychiatric Interview (M.I.N.I.) [32] was carried out to identify participants with DSM-IV and ICD-10 psychiatric disorders. Demographic data (age, gender, relationship status, educational level, and occupation), health data (somatic disorders, medication, mental disorders, and mental disorders in first-degree relatives), and data about the participants’ work history (hospital ward they working in at the time of data collection, duration of working in the hospital ward, and duration of working in healthcare) were collected.

The MOX3 ECG-Actigraph device (Maastricht Instruments, Maastricht, The Netherlands) was used to record ECG and motor activity. This is a non-invasive single-channel ECG device that simultaneously registers a participant’s motor activity [33].

The Biofeedback application was used to help reduce participants’ occupational stress during working hours [34]. The application consists of two interfaces:

(1) An interface for the measurement of the unique resonance frequency, containing a continuously pulsating circle that guides the participant’s respiration rhythm (upper right corner of the screen); provides information about the breathing frequency, the duration of the measurement, the last-measured resonance frequency; has a start/stop button (left upper corner of the screen); and shows the participant’s heart rate curve (lower part of the screen)
(2) An experiment interface was used that provides instructions during the intervention (HRV BF training) and under control conditions (habitual relaxation), contains the five-item State Scale of Short Version of the Spielberger State–Trait Anxiety Inventory (STAIS-5) [35] and one-item Distress Thermometer [36] questionnaires designed to evaluate the perceived stress level. The interface of the experiment during HRV BF training is very similar to the interface for the measurement of the resonant frequency. It contains information about the participants’ unique resonance frequency and information about how successfully the participants approached or reached their unique resonance frequency (top of the screen), a pulsating circle that guides the participant’s respiration rhythm (left center of the screen), information about the duration of the measurement, a start/stop button (right center of the screen), and information about the correct use of the abdominal breathing technique (bottom of the screen).

#### 2.1.3. Procedure

The Mini International Neuropsychiatric Interview [32] was carried out first; then, the participants completed the demographic questionnaires. MOX3 devices were attached to the participants, and they were worn during the entire experiment (five days), continuously. Then, the participants were shown how to use the abdominal breathing technique that was employed when measuring the unique resonance frequency and during HRV BF training. After learning the correct breathing technique, the unique resonance frequency was measured with the Biofeedback application. Following the measurement of the resonance frequency, the participants used the Biofeedback application during breaks at work in a quiet and separated examination room. The Biofeedback application ran on a tablet, and a pulse oximeter was connected to the tablet to continuously monitor the heart rate of the participant. After the participant signed in, the Biofeedback application asked the participants to measure their own individual resonance frequency. For the measurement of the resonance frequency, the application instructed the participant to breathe for one minute at each of the following frequencies: 16, 14, 12, 10, 9, 8, 7.5, 7, 6.5, 6, 5.5, and 5 breaths per minute. The application guided the participant’s respiration rate through a continuously pulsating circle (inhale when narrowing, exhale when expanding). After measuring the unique resonance frequency, the participants used the Biofeedback application during work breaks. All the participants received both HRV BF and habitual relaxation training, and the order of the conditions was in a fixed alternating sequence.

Before the HRV BF training or habitual relaxation, the Biofeedback application instructed the participant to move freely in the examination room for one minute; then, the participant evaluated their baseline perceived stress level on the STAIS-5 [35] and the Distress Thermometer [36]. After the evaluation of the perceived stress level, the participant received either the HRV BF training or, under controlled conditions, the habitual relaxation. During the HRV BF training, the participant was instructed to breathe near or at their resonance frequency level. Under controlled conditions, the Biofeedback application instructed the participants to spend their time freely by reading news, social media, etc., on their mobile phones. Both HRV BF training and habitual relaxation lasted for six minutes, and they were followed by an evaluation of the perceived stress level on the STAIS-5 [35] or the Distress Thermometer [36]. After the determination of the stress level, the participants were instructed to move freely for one minute in the room. After one minute, the Biofeedback application told the participant that the assessment was over.

### 2.2. Study 2: Stress Induction

#### 2.2.1. Participants

Ten participants from the Faculty of Science and Informatics of the University of Szeged were recruited for this study. One of the participants was excluded due to improper data. After data cleaning, the sample consisted of nine participants, all of whom were males with a mean age of 27.56 years (SD = 7.81, range: 21.00–46.00). No participant suffered from any mental disorders according to the MINI Interview. The local ethics committee at the National Public Health Center (15239-5/2023/EUIG) approved the study. Prior to participating in the study, the participants were requested to sign an informed consent form. The participants did not receive any remuneration for taking part in the study.

#### 2.2.2. Data Collection

The MINI International Neuropsychiatric Interview [32] was used for screening for the presence of mental disorders. Demographic and health data sets were collected via a questionnaire. The State Scale of the Hungarian version of STAI (STAI-S) [37] and Distress Thermometer [36] questionnaires were used for the assessment of perceived stress level.

Electrophysiological instruments were used to measure galvanic skin response, blood pressure, pulse, and respiration. Also, ECG signal and motor activity were recorded with an MOX3 ECG-Actigraph [33].

Stress was induced in a within-subject design, using two stress induction methods, namely the Socially Evaluated Cold Pressor Test (SECPT) [31] and the Socially Evaluated Stroop Test (SEST). The Socially Evaluated Cold Pressor Test is an effective stress induction method, developed by Schwabe et al. [31]. During the SECPT experiments, the participants immersed their hand in a bowl of ice-cold water (0–4 °C) for three minutes, while the researcher observed the participants’ behavior and recorded it. The participants’ behavior was also recorded by a video camera, and at the same time the video recording was displayed to the participant. The researcher maintained distant and objective behavior toward the participant, and did not provide any positive nonverbal or verbal reinforcement. The social elements of the SECPT experiment (distant behavior of the researcher, no positive feedback, video recording, observation of the participant’s behavior) enhanced the distress caused by the immersion of the participant’s hand in the ice-cold water [31,38].

The Socially Evaluated Stroop Test (SEST) is a novel stress induction method developed by our research team. The SEST method integrates the incongruent phase of the computerized version of the Stroop test [39] and the social elements of SECPT [31]. In the incongruent phase of the Stroop test [39], the participant sees words of colors (“blue”, “yellow”, “green”, and “red”) written with different colors of ink, where the color of the ink does not match the meaning of the word. The words are displayed on the computer, four different buttons of the keyboard represent the color names, and the participant’s task is to determine the color of the word by pressing down the button that corresponds the color of the word. To enhance the stress response, social elements from SECPT [31] were incorporated into the SEST. In this approach, the participants’ behavior and performance are observed and recorded by a researcher; furthermore, a video recording is simultaneously made and displayed to the participant. The researcher avoids providing any positive verbal or nonverbal reinforcement to the participant. The participant is told that the number of their correct answers and their reaction time will be analyzed too, so it is necessary to provide correct answers quickly. To provide feedback to participants about their performance, an annoying sound signal occurs when the participant gives an incorrect answer or does not respond.

#### 2.2.3. Procedure

A demographic questionnaire was completed and MOX3 devices were attached to the participants in the morning, at least four hours before the stress induction session. The participants were also asked to walk up three flights of stairs. Physical activity was necessary for calculating the Master Curve. The examination continued using one of the stress induction methods on the afternoon of the same day, and with the other stress induction method on the afternoon of the other day. The design of Study 2 was crossover; hence, the sequence of the stress induction methods was randomized (50% of the sample started with SECPT [31], and the other 50% started with SEST). Each participant received both stress induction methods on different days at the same time in the afternoon, between 12 a.m. and 8 p.m., to minimize the effect of cortisol fluctuation on the circadian rhythm. After arriving at the laboratory, the electrophysiological instruments were attached to the participants, and the participants were asked to sit quietly for 15 min before the stress induction session. In order to determine the baseline level of the perceived stress, the Hungarian version of STAI-S [37] and the Distress Thermometer [36] were completed 14 min after their arrival. After the questionnaires were completed, the participants either received the SEST method or the SECPT [31] method, depending on the order of the randomization. Immediately after the stress induction session, the participants completed the STAI-S [37] and Distress Thermometer [36] questionnaires. As the questionnaires were completed, the researcher informed the participant that the stress induction was over. The stress induction phase was followed by a twenty-minute recovery phase, when the participant was asked to sit quietly for 20 more minutes. At the end of the twenty-minute recovery phase, the participants completed the STAI-S [37] and Distress Thermometer [36] questionnaires. The Mini International Neuropsychiatric Interview [32] was conducted after the second stress induction session.

### 2.3. Evaluation

Among the heart rate variability parameters, HR, SDNN, and RMSSD were examined as the most frequently used time-domain parameters (Note that RMSSD can be derived from nonlinear, Poincaré-plot-based analyses, as well). In addition to these, we introduced another parameter, called the normalized variability ”LnSD”, which will be described later.

Practically all the parameters that characterize instantaneous heart rate variability (i.e., not considering the time-averaged measures) are heart rate-dependent, irrespective of being time-domain, frequency-domain, or nonlinear ones [5,6,7,8,9,10,11,12,15,21]. In general, this dependence is extremely decisive, since usually a double-exponential function describes the relationship between such a typical HRV parameter as RMSSD and the heart rate [15]. If this dependence is not accounted for properly, the variability parameter will mainly reflect the changes in the heart rhythm when examining the phenomena, and no independent physiological meaning can be ascribed to it. To overcome this problem, the various variability parameters should be normalized to HR. To this end, here we introduced a normalized variability parameter, based on the heart rate dependence of the heart rate variability parameter RMSSD. We used the “Master Curve” (MC), introduced in [15], for normalization, as follows.

**Figure 1:**
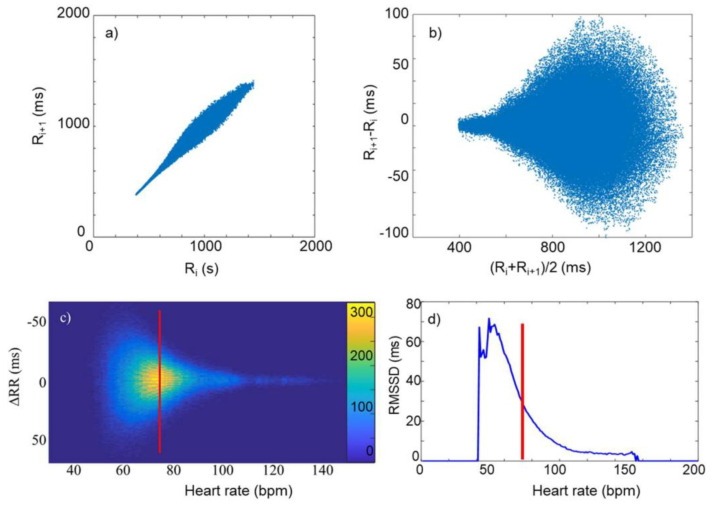
(**a**) Poincaré plot of a typical RR time series. (**b**) The same in Bland–Altman-like representation. (**c**) ΔRR as a function of heart rate (mHR), as calculated from the data in (**b**). The color code shows the frequency of the data. (**d**) The RMSSD versus HR curve (MC), determined from data in (**c**), as the RMS of the distribution of ΔRR values at each HR. The red lines stand for illustration of the way of calculation at an ad hoc HR value. (Reproduced from Búzás et al. [15]).

Stated briefly, the determination of the MC started from the well-known Poincaré plot of the RR time series, where the X and Y coordinates of the i-th point on the plot are R_i_ and R_i+1_, respectively, where R_i_ represents the length of the i-th beat-to-beat interval (Figure 1a). The subsequent Bland–Altman type of representation ((R_i_ + R_i+1_)/2, R_i+1_ − R_i_) results in a point set quasi-symmetric to the abscissa (Figure 1b), which, after transformation from the RR scale to the HR scale and fitting Gaussians to the ΔRR distribution at each effective HR (Figure 1c), gives a good-quality determination of an HRV(HR) function, in the form of RMSSD as a function of HR, called the MC (Figure 1c).

#### Normalization of HRV

To be able to determine the MC, the MOX3 device was worn for several hours while performing daily activities, and we asked the applicants to perform a slightly more intense movement (e.g., climbing stairs), in order to obtain a Master Curve over as wide an HR range as possible. This provided a good reference curve for normalization, and the actual dRR values determined during the relaxation or stress induction tests were normalized using the MC:

nSD = SD(HR)/MC(HR)

Lastly, we took the standard deviation value of these normalized values for 60 s-long windows. Since the nSD values obtained in this way showed a lognormal distribution with a spread around 1, we took its logarithm, so the mean value became 0. Negative values indicate the cases where the variability is below the reference curve, while positive values indicate the cases where the variability is above the reference curve:

LnSD = log(StdDev(nSD,60))

The Kolmogorov–Smirnov test was used to determine the statistical significance. Before, during, and after applying the stress-alleviating (HRV BF training or habitual relaxation) and stress induction methods (SECPT [31] or SEST), we extracted a section of each and compared the distribution of the parameters within the section.

## 3. Results

### 3.1. Changes in Heart Rate Variability During Relaxation

**Figure 2:**
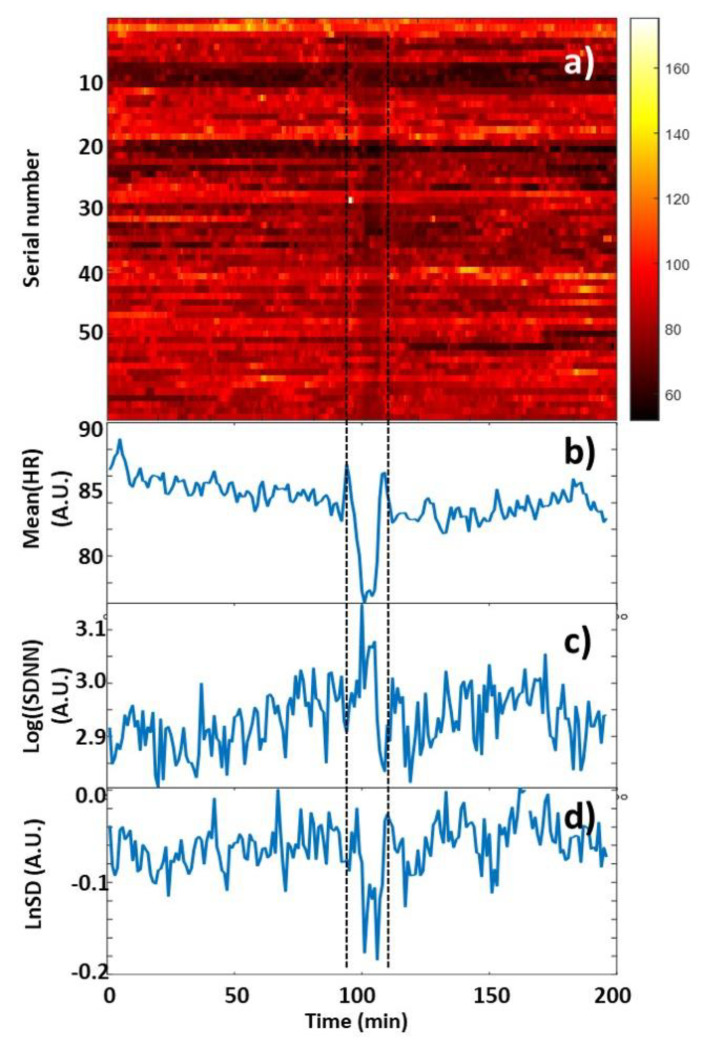
(**a**) Map of the HR time series (90 tachograms), synchronized to the start of the relaxation process. The HR values are color-coded. (**b**) The averaged tachogram of all the measurements. (**c**) Log(SDNN) and (**d**) LnSD time series, as averaged over each measurement.

**Figure 3:**
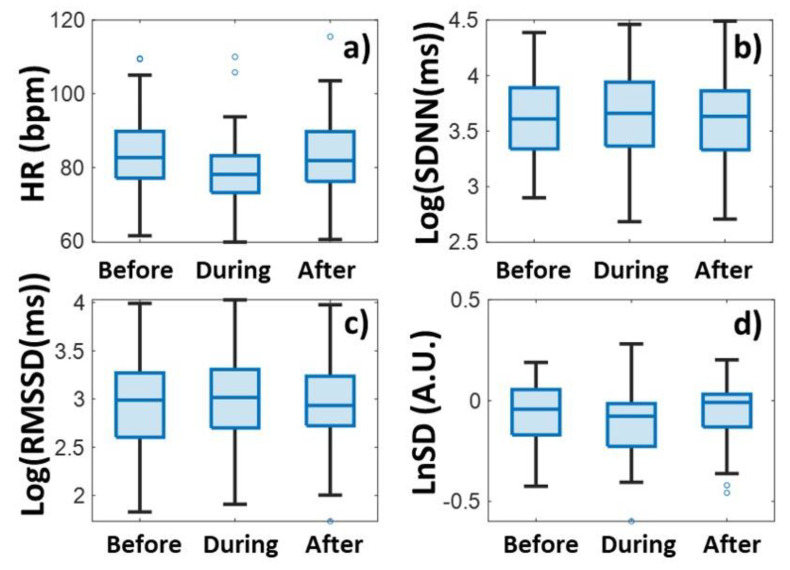
Heart rate (HR) (**a**) and heart rate variability (SDNN (**b**), RMSSD (**c**), and LnSD (**d**)) data before, during, and after HRV-biofeedback training (HRV BF), respectively. The blue dots stand for the outlier points.

**Figure 4:**
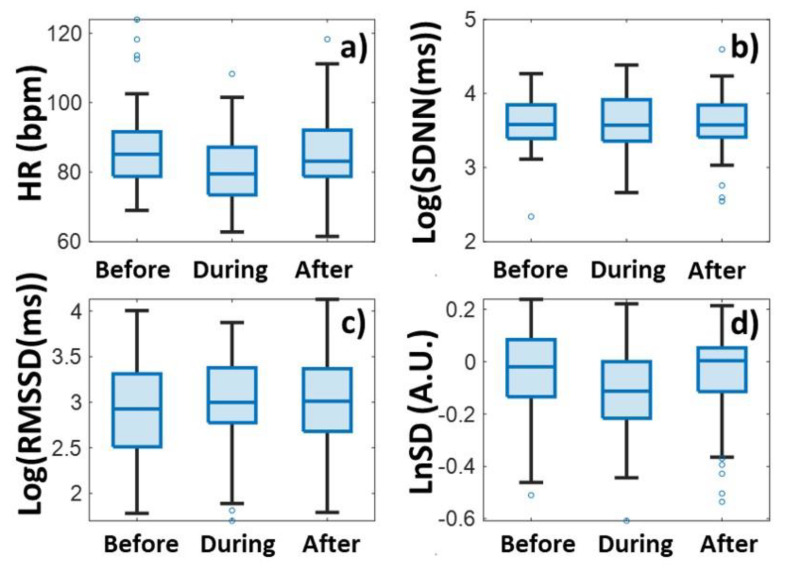
Heart rate (HR) (**a**) and heart rate variability (SDNN (**b**), RMSSD (**c**), and LnSD (**d**)) data before, during, and after habitual relaxation (RLX), respectively. The blue dots stand for the outlier points.

Heart rates were plotted in a color-coded map by clipping 100 min before, during, and after the relaxation sessions for each participant, and synchronizing the data to the onset of relaxation (Figure 2a). The averaged tachogram of all measurements is shown in Figure 2b, while the time series of two HRV measures, Log(SDNN) and LnSD, are averaged over each measurement (Figure 2c and Figure 2d), respectively. The average and the standard deviation of the pulses for all persons are shown in Figure 3a and Figure 4a for biofeedback training and habitual relaxation, respectively. In the case of both relaxation methods, the heart rate averaged over the group was decreased by almost the same amount, by approximately by ~7 bpm. The change is significant at *p*~0.01.

In the case of SDNN parameters, the picture is somewhat more complex when examining the two types of relaxation parameters. While in the case of HRV BF, the SDNN tends to show a rising trend (Figure 3b), in the case of habitual RLX-type relaxation it fluctuates strongly, after which it returns to the baseline (Figure 4b).

Figure 3c and Figure 4c show the RMSSD variability parameter values. Even though the RMSSD parameter fluctuates quite a lot, a definite increase is observed during both relaxation sessions. The extent of the protrusion compared to the surrounding baseline is around 0.1–0.2 for both relaxation methods, measured on a logarithmic scale.

In contrast, the normalized variability parameter clearly decreases due to the relaxation events, where the decrease is between −0.1 and −0.2 on a logarithmic scale for both relaxation methods (Figure 3d and Figure 4d).

An examination of the significance levels provides an interesting result (Table 1). The variation in HR is significant (*p* ~ 0.01) for both relaxations, but with the change in non-normalized variabilities, the significance is very low (between *p* ~ 0.5 and 0.8), unlike the normalized parameters, where the difference is also significant (*p* < 0.2). These results also tell us that the normalized HRV parameter can be more useful for detecting changes than the non-normalized ones. (We will elaborate on this issue in the Discussion section later on.) There is no significant difference between the two types of relaxation methods in either the heart rate or the variability parameter values (Table 1). Both relaxation methods have approximately the same effect on the change in variability parameters.

### 3.2. Heart Rate Variability During Stress Induction

**Figure 5:**
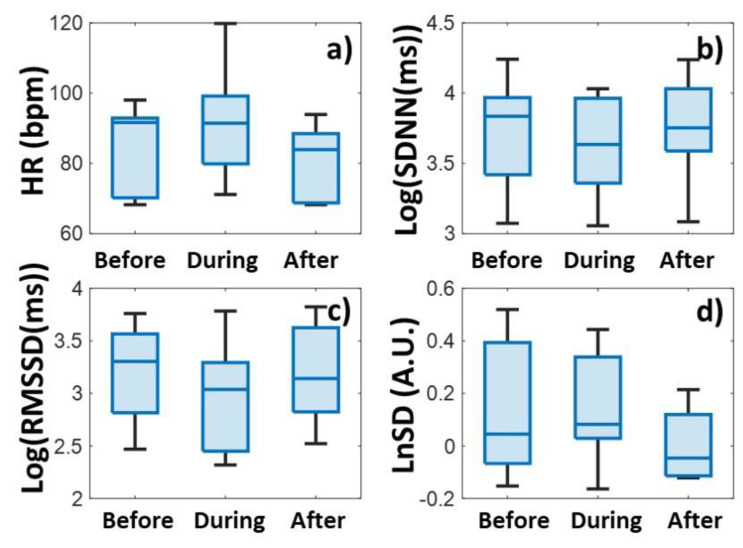
Heart rate (HR) (**a**) and heart rate variability (StdDev (**b**), RMSSD (**c**),and LnSD (**d**)) data before, during, and after the Socially Evaluated Stroop Test (SEST), respectively.

**Figure 6:**
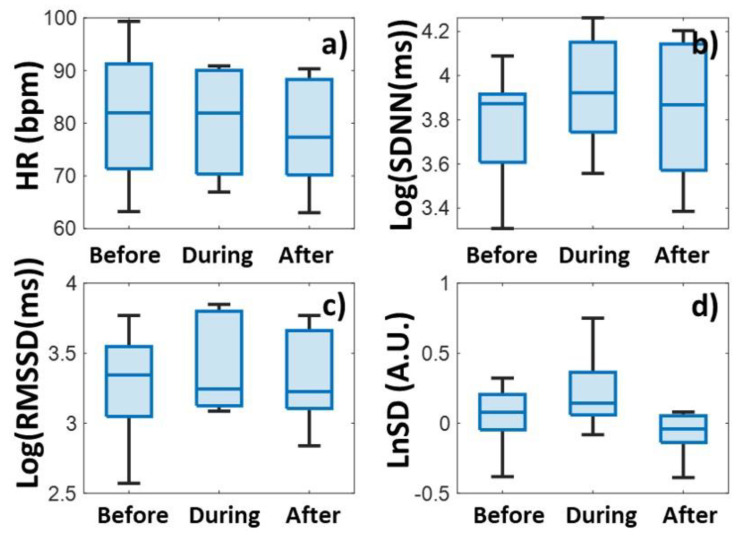
Heart rate (HR) (**a**) and heart rate variability (StdDev (**b**), RMSSD (**c**), and LnSD (**d**)) data before, during, and after the Socially Evaluated Cold Pressor Test (SECPT), respectively.

In the case of SEST-type stress induction, the heart rate increased by an average of 7 bpm, as can be seen in Figure 5a and in Table 2 (*p* = 0.25), but there was no significant heart rate change in the case of SECPT [31] (Figure 6a, *p* = 0.92). However, after the stress induction sessions, with both types of stress induction, the heart rate returned to a lower value.

Although the SDNN value decreased during the stress induction session of the SEST type, the decrease was not significant (*p* = 0.95), and it returned to the initial value after the stress induction session. In the case of SECPT [31], the SDNN increased (*p* = 0.51) and then returned to a slightly higher value than the initial value (Table 2).

By contrast, the normalized variability increased in the case of both stress induction sessions. The increase was higher in the case of SECPT [31]. Curiously, the normalized variability was higher even before the stress induction than after the cessation of the stress.

## 4. Discussion

### 4.1. Stress-Alleviating Measurements (Heart Rate Variability Biofeedback Training and Habitual Relaxation)

On comparing the stress-alleviating methods, we found that there was no significant difference between the results in the two cases. Both the increase in heart rate and the change in HRV are similar. Although the exact time information is not visible in the figures above, the effect of HRV BF, in terms of variability, seems to be stronger, since it returns to the baseline a few minutes later than habitual RLX-type relaxation.

### 4.2. Stress Induction Measurements (SECPT and SEST)

Upon examining the variability parameters, we noted a similar change in the normal variability parameter values in both stresses, but the heart rate increased significantly only in the SEST case, and there was no detectable change in the heart rate in the SECPT [31] case. With SECPT [31], the variability parameters returned to the baseline value almost immediately after the end of the stress session, while with SEST several minutes were required. From this, we conclude that both stress-inducing methods are suitable for inducing stress, but the response to SEST stress was greater, and the state normalized more slowly.

The heart rate parameters obtained with the standard evaluation follow the same patterns as those previously reported in the literature [22,23]. The heart rate increases during the SEST session, while there is no detectable change in it in the SECPT [31] session. The values of the SDNN and RMSSD parameters decrease as a result of the stress induced during the SEST session. Despite this, with SECPT [31], both parameters follow an upward trend. As for the statistical significance levels, with the standard evaluation, we cannot see any significant change, in sharp contrast with the new evaluation with normalized parameters, where the effects are significant.

### 4.3. Comparison of Stress-Alleviating Measurements and Stress Induction Measurements

On comparing the stress-alleviating methods with those of stress induction, we notice that both the pulse and the variability do change consistently. The heart rate—as expected in the case of relaxation—decreases, but it increases in the case of stress. Although we also determined the parameters characterizing traditional variability, they apparently do not contain any new information, but simply follow the trend dictated by the heart rate: they have a decreasing value with an increasing pulse, and an increasing value with a decreasing pulse. Still, normalized variability can be used as an HR-independent parameter, as it can reflect changes even in cases where there is no significant heart rate change. It also changes consistently in the sense that the normalized variability increases with stress and decreases with relaxation. This is a surprising finding after reviewing the results published earlier in the literature.

### 4.4. Comparison with Previously Reported Results in the Literature

The conventional evaluation of our stress and relaxation experiments is consistent with the earlier results published in the literature. The standard evaluation gives an increasing variability for relaxation [17] and decreasing variability for stress, as found in the literature [23]. In contrast, however, the normalized variability defined by us decreased in both relaxation sessions and increased in both stress sessions.

Why is there a difference between the results of the standard evaluation and the normalized parameters?

The answer to this question comes from the heart rate dependence of the variability parameters. All the parameters characterizing the heart rate variability strongly depend on the heart rate. In the case of SDNN and RMSSD, this dependence is exponential-like [15], which is manifested in the fact that both parameters decrease exponentially as the heart rate increases. This means that even with a small change in heart rate there will be a large change in the variability parameter value. However, in the literature, the change in heart rate is usually not taken into account when interpreting the change in variability, hindering the comparison of results measured at different heart rates. If, however, the change in heart rate is taken into account, the results published in the literature can be immediately brought into line with the current ones. For example, in the case of stress, the variability decreases due to the increasing heart rate, which is simply a consequence of the heart rate change. Similarly, during relaxation, the heart rate decreases, and the resulting increase in variability is only the result of the change in heart rate.

The SECPT [31] test gave different results because there was no change in the heart rate in that case, so the pulse dependence of the variability parameters did not give rise to any interpretation problems.

**Figure 7:**
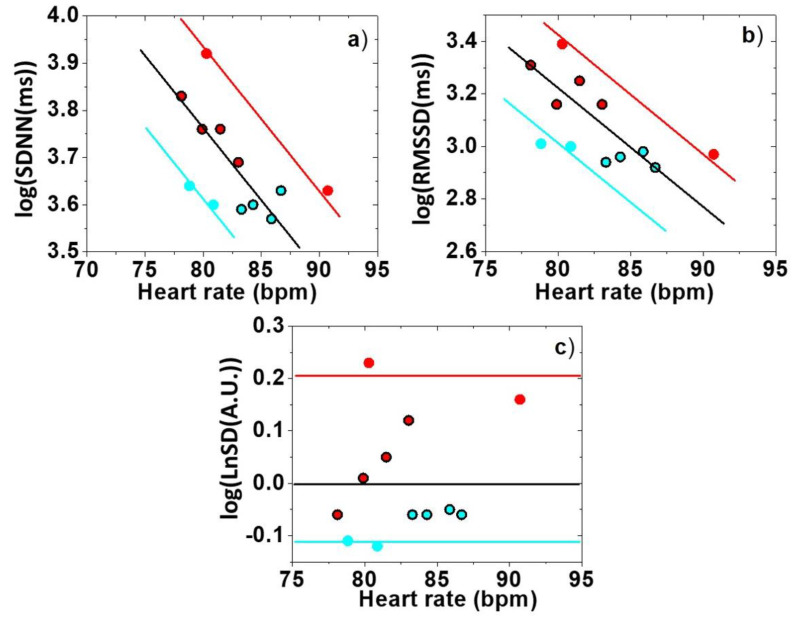
A Cumulative summarizing diagram of the effects of stress and relaxation on conventional (SDNN (**a**) and RMSSD (**b**)) and normalized (LnSD) (**c**) HRV parameters. Red—stress induction; cyan—relaxation; black—reference (before and after stress or relaxation).

To clarify what we are talking about, all the data values presented in Table 1 and Table 2 have been plotted on a single graph in such a way that the SDNN, the RMSSD, and the normalized variability, LnSD, are presented as a function of the heart rate (Figure 7). In the case of SDNN, it can be seen that the data from different measurements show the same trend, and there is a well-defined baseline. The vertical axis is logarithmic, so when plotted in the semi-logarithmic plot, we obtain just a straight line, which is consistent with the fact that the heart rate dependence is exponential. In this plot, for both types of stress, SDNN values are shifted upward from the baseline, even for SEST, which is exactly the opposite of what the standard assessment would predict. Also, in the relaxation experiments, the variability just moves downwards, in contrast with the standard evaluation.

The change in heart rate variability can be properly interpreted if the variability parameters are examined as a function of the heart rate. Namely, the HRV(HR) function should be considered (such as the MC in [15]), and the changes compared to it should be examined.

The above representation describes the changes in a physiological sense as well. The baseline is well defined. As a result of stress, the variability moves upwards, while as a result of relaxation, it moves downwards. The reason why it displays changes in the opposite direction compared to the picture developed so far is simply that in our evaluation we took into account the change in the baseline (Figure 7).

The physiological interpretation of variability parameters also must be similarly modified. Until now, there have actually been trivial physiological conclusions derived from the increase in heart rate variability, such as the reduction in sympathetic activity and the increase in parasympathetic activity, due to a decrease in heart rate during relaxation. However, this interpretation is problematic because the change in the heart rate inevitably implies a change in HRV, so the conventional description simply assigns the actual state of the sympathovagal balance to the change in the pulse. If, however, we also consider the change in the baseline, then the real question is, as Figure 7 suggests, what physiological changes take place when the variability shifts up or down compared to the baseline?

During daily routine conditions, the HRV fluctuates according to the momentary physical activity and mental state. The MC, i.e., the RMSSD(HR) function derived from a several-hour-long period, represents the mean sympathovagal balance, which proved to be conservative for an individual on the weekly/monthly scale. Hence, the momentary changes in the normalized variability (LnSD) can be interpreted as a consequence of the actual deviations from the mean sympathovagal balance. Negative deviations from the baseline may indicate inhibitory effects, while positive deviations may indicate excitatory effects on the autonomic nervous system components, e.g., as a consequence of ice treatment during SECPT [31], the LnSD increases, while the HR does not change significantly. Here, we hypothesize that the HR-increasing effect of the stimulation of the sympathetic nervous system is counterbalanced by a corresponding increase in the parasympathetic activity.

### 4.5. Limitations

In this study, we examined only moderate stress and relaxation effects [15], which can be detected in HRV. However, more significant stress effects might reveal different trends. We could also increase the sample size to obtain a better statistical basis of the result to be obtained.

So far, we have investigated only one HRV parameter, the logarithm of the normalized SD, i.e., LnSD. This is suitable for showing whether the examined person is in an excited or inhibited state. A more accurate mapping of the patient’s psychosomatic state nevertheless requires the inclusion of additional parameters conveying independent information. It should be mentioned that almost all the HRV parameters are predominantly heart rate-dependent, so a proper normalization of the other HRV parameters based on the heart rate will also be necessary in the future. Here, we note that a similar procedure is required for the description in the frequency-domain parameters (LF and HF components). In addition, due to the Parseval rule there is a strong connection between the characteristics of the time-domain (RMSSD, SDNN) and Fourier components.

## 5. Conclusions

The HR-normalized RMSSD parameter appears suitable for characterizing the patients’ SEST and SECPT [31]/relaxation state (HRV BF, habitual relaxation), and it is sensitive and robust even to restrained effects. While in our studies neither of the applied stress and relaxation level exceeded the moderate scale, their effects could still be clearly detected with the help of this single parameter, which does not reflect the trivial changes in HR but contains information independent of the heart rate. Our procedure based on normalized HRV measurements, therefore, is envisaged to be used as a sensitive stress indicator in the future, and to help reveal some of the complex effects of the autonomic nervous system on the cardiac system in general.

## Figures and Tables

**Table 1:** Averaged heart rate and heart rate variability (SDNN, RMSSD, and LnSD) data before, during, and after HRV biofeedback training (HRV BF) and habitual relaxation (RLX), respectively, and the significance of deviations (*p*; * *p* ≤ 0.05; ** *p* ≤ 0.01; *** *p* ≤ 0.001).

	Heart Rate Variability (HRV) Biofeedback					Habitual Relaxation (RLX)				
	Before	During	After	Before–During *p*	During–After *p*	Before	During	After	Before–During *p*	During–After *p*
Mean (HR), bmp	84.28	78.82	83.29	0.01 **	0.02 *	86.69	80.87	85.87	0.00 ***	0.02 *
Log(SDNN), ms	3.60	3.64	3.59	0.80	0.81	3.63	3.60	3.57	0.82	0.82
Log(RMSSD), ms	2.96	3.01	2.94	0.66	0.51	2.92	3.00	2.98	0.53	0.82
LnSD (A.U.)	−0.06	−0.11	−0.06	0.19	0.01 **	−0.06	−0.12	−0.05	0.03 *	0.01 **

**Table 2:** The averaged heart rate and heart rate variability (SDNN, RMSSD, and LnSD) data before, during and after the Socially Evaluated Stroop Test (SEST) and the Socially Evaluated Cold Pressor Test (SECPT), respectively, and the significance of deviations (*p*, * *p* ≤ 0.05), as determined from the Kolmogorov–Smirnov test.

	Socially Evaluated Stroop Text (SEST)					Socially Evaluated Cold Pressor Text (SECPT)				
	Before	During	After	Before–During *p*	During–After *p*	Before	During	After	Before–During *p*	During–After *p*
Mean (HR), bmp	83.03	90.72	79.90	0.25	0.25	81.47	80.28	78.11	0.92	0.92
Log(SDNN), ms	3.69	3.63	3.76	0.95	0.60	3.76	3.92	3.83	0.51	0.51
Log(RMSSD), ms	3.16	2.97	3.16	0.60	0.60	3.25	3.39	3.31	0.51	0.51
LnSD (A.U.)	0.12	0.16	0.01	0.60	0.08	0.05	0.23	−0.06	0.51	0.05 *

## Data Availability

Data are available from the first/corresponding authors on reasonable request.

## Abbreviations

RR	Time interval between successive R peaks of ECG recordings
HR	Heart rate (beats per minute)
HRV BF	Heart rate variability biofeedback
RLX	Habitual relaxation
SEST	Socially Evaluated Stroop Test
SECPT	Socially Evaluated Cold Pressor Test
HRV	Heart rate variability
StdDev	Standard deviation
SDNN	Standard Deviation of Normal-to-Normal interbeat intervals measured in milliseconds
SD	Successive differences in RR intervals
RMSSD	Root Mean Square of Successive Differences between normal heartbeats
MC	Master Curve
nSD	SD values normalized by MC
LnSD	Logarithm of the standard deviations of nSD
HR	Heart rate
HF	High-frequency component
LF	Low-frequency component
SNS	Sympathetic nervous system
HPA	Hypothalamic–pituitary–adrenal
ANS	Autonomic nervous system
